# ASSOCIATED FACTORS OF PARTICIPATION IN DIGITAL TECHNOLOGY: A STUDY BASED ON OLDER PEOPLE IN CHINA

**DOI:** 10.1093/geroni/igad104.3187

**Published:** 2023-12-21

**Authors:** Lan Liu, Chao Guo, Liangshu Qi, Yingying Wang, Ruixin Shu

**Affiliations:** Institute of Population Research, Peking University, Beijing, Beijing, China (People’s Republic); Peking University, Beijing, Beijing, China (People’s Republic); School of Economics and Management, Beijing, Beijing, China (People’s Republic); Institute of Population Research, Peking University, Beijing, Beijing, China (People’s Republic); Institute of Population Research, Peking University, Beijing, Beijing, China (People’s Republic)

## Abstract

There has been an increasingly important trend that more and more older people take part in activities of digital technology nowadays in China. In this study, the research question associated to determinants of older people’s digital engagement was addressed, which has been quantitatively analyzed by statistical models in a sample of older people 60 to 98 years of age from China Longitudinal Aging Social Survey (N=11207). For dependent variable, digital participation of older people has been measured by the access to the Internet with mobile phones and various digital devices, whereas for independent variables, individual, situational, environmental, and social policy factors which were based on a conceptual model of productive aging framework have been constructed to present academic explanations on dependent variable. Results of Probit and Ordered-Probit regression showed that the older people’s participation in digital technology was closely related to individual and situational factors, whereas the effects of environmental factors and social policies were not statistically significant. Older adults who were much younger and physically healthier had higher probability of participation in digital technology. Meanwhile, those who lived in urban area, had higher levels on income, literacy and social support were more likely to be involved in digital activities. Conclusions shed light on the significance of academic research to improve productive ageing and successful ageing which takes a more active role on exploring and grappling with issues related to application of digital technology especially technological difficulties that older people have confronted with in an ageing society.

